# “Inverse signaling” of the transmembrane chemokine CXCL16 contributes to proliferative and anti-apoptotic effects in cultured human meningioma cells

**DOI:** 10.1186/s12964-016-0149-7

**Published:** 2016-10-27

**Authors:** Kirsten Hattermann, Kareen Bartsch, Henrike H. Gebhardt, H. Maximilian Mehdorn, Michael Synowitz, Anne Dorothée Schmitt, Rolf Mentlein, Janka Held-Feindt

**Affiliations:** 1 Department of Anatomy, University of Kiel, Otto-Hahn-Place 8, 24118 Kiel, Germany; 2 Department of Neurosurgery, University of Schleswig-Holstein Medical Center, Campus Kiel, Arnold-Heller-Str.3, Building 41, 24105 Kiel, Germany

**Keywords:** Chemokines, Chemokine receptors, Cellular communication, Meningioma, Inverse signaling

## Abstract

**Background:**

Chemokines and their receptors play a decisive role in tumor progression and metastasis. We recently found a new signaling mechanism in malignant glioma cells mediated by transmembrane chemokines that we termed “inverse signaling”. According to this hypothesis, soluble (*s*)*-*CXCL16 binds to the surface-expressed transmembrane (*tm) -*CXCL16, and induces signaling and different biological effects in the stimulated cells, so that the transmembrane ligand itself acts as a receptor for its soluble counterpart. Now, we hypothesized that “inverse signaling” via *tm-*CXCL16 might also take place in meningiomas, a completely different, benign tumor entity.

**Methods:**

We used quantitative reverse-transcription polymerase chain reaction, immunocytochemistry and western blot to detect CXCL16 and CXCR6 in human meningioma cells isolated from 28 human meningiomas. Subsequently, we stimulated cultured human *tm-*CXCL16-positive, CXCR6-negative meningioma cells with recombinant *s-*CXCL16 and analyzed binding, signaling and biological effects using RNAi silencing to verify specificity.

**Results:**

In fact, cultured human meningioma cells considerably express CXCL16, but substantially lack CXCR6, the only known CXCL16 receptor. These receptor-negative cells could bind *s-*CXCL16, and responded to *s-*CXCL16 application with activation of the intracellular kinases ERK1/2 und Akt. As a consequence, we observed increased proliferation and rescue of apoptosis of cultured meningioma cells. Since binding and signaling were abolished by siRNA silencing, we concluded that *tm-*CXCL16 specifically acts as a receptor for *s-*CXCL16 also in human meningioma cells.

**Conclusion:**

These findings underline our recent report on the mechanism of inverse signaling as a broad biological process also observable in more benign tumor cells and contributing to tumor progression.

## Plain english summary

Cells communicate by ligands that bind to their respective receptors. Some ligands are transmembrane molecules which means they span through the cell membrane. The part of these ligands at the outer cell surface can be liberated from its membrane stack yielding a soluble ligand that is present outside of the cell and can meet corresponding receptors at the surface of the same or another cell. Some tumor cells have high levels of transmembrane ligands but do not produce the corresponding receptors for these molecules. Serendipitously, in malignant brain tumor cells we detected a novel, alternate mechanism of cell communication which we term “inverse signaling”: Here, a transmembrane ligand (namely transmembrane chemokines) acts as a “receptor” for its soluble counterpart. By studying now benign human tumor cells derived from the linings of the brain and spinal cord, so called meningiomas, Hattermann et al. show that the soluble form of the transmembrane chemokine CXCL16 binds to its transmembrane equivalent in these tumor cells, too. This interaction initiates intracellular signaling pathways that promote cell growth and make the meningioma cells more resistant to cell death. Thus, Hattermann et al. showed that the “inverse signaling” paradigm also takes place in benign tumor cells, suggesting that it helps to fine-tune the communication between cells as a broad biological process.

## Background

The chemokine CXCL16 (synonym SR-PSOX) was originally discovered as a scavenger receptor for oxidized LDL [1], and independently as a ligand for the CXC-chemokine receptor CXCR6, also termed Bonzo, TYMSTR, STRL33 [2]. CXCL16 is synthesized as a transmembrane *(tm)* multi-domain molecule consisting of a chemokine domain followed by a glycosylated mucin-like stalk, a single transmembrane helix and an intracellular tail. However, a soluble (*s-*)CXCL16 form can be generated by constitutive or induced cleavage from the transmembrane form by the cell-surface proteases ADAM10 and 17 (ADAM, a disintegrin and metalloproteinase) [3, 4].

In previous investigations we and others determined that CXCL16 is definitely involved in tumor progression of different tumor types, e.g. gliomas, schwannomas, lung and breast tumors [5–9]. However, apart from its classical signaling mode in which the proteolytically released chemokine domain would bind to and signal via its receptor CXCR6, we recently discovered an alternate signaling mechanism for transmembrane chemokines in glioma cells which we termed “inverse signaling” [10]. According to this novel mechanism, the proteolytically released (or also recombinant) *s-*chemokine binds to an intact transmembrane chemokine specimen inducing intracellular signaling and biological effects independently from the expression of the receptor CXCR6. We initially demonstrated this mechanism in cultivated malignant human glioma cells, from which we had previously shown to express high levels of CXCL16, but a lack of the corresponding receptor CXCR6 [11].

Apart from that, we could show that solid human meningiomas - the second most common intracranial tumors - are also characterized by high expression levels of CXCL16 while CXCR6 expression is more restricted [12]. Meningiomas develop from arachnoid cap cells, and although malignant variants exist (atypical meningiomas, World Health Organization, WHO grade II and anaplastic meningiomas, WHO grade III) most meningiomas (about 90 %) are slowly growing benign (WHO grade I) tumors [13, 14].

Facing the high CXCL16 expression levels in human meningiomas, we raised the question if “inverse signaling” of CXCL16 previously observed in highly malignant glioma cell might also take place in benign meningioma cells. Therefore, we analyzed binding, intracellular signaling effects and different biological readouts in cultured primary human meningioma cells upon stimulation with *s*-CXCL16. This should validate our recent hypothesis on inverse signaling in more benign brain tumor cells as a broader biological process.

## Methods

### Tumor specimens

Meningioma samples were surgical dissected tissues from the Department of Neurosurgery (Kiel, Germany) and were obtained in accordance with the Helsinki Declaration of 1975 with approval of the ethics committee of the University of Kiel, Germany (file reference: D 442/11) after written consent of donors. Tumors were classified according to the WHO criteria into the various subtypes of meningiomas [13]. The diagnosis was established by a pathologist. In this study, a total number of 28 meningiomas were included (P1 to P28). With exception of P10, P25, P27 and P28 all meningiomas were classified into WHO grade one, P10, P25, P27 and P28 were grad two meningiomas. If possible (enough material available), for different investigations matched probes of individual tumor samples were used (see Table 1).

**Table 1:** Documentation of patients’ samples and use for experiments

Sample	WHO grade	Expression analysis qRT-PCR/ICC/WB	Kinase-WB	Binding	Proliferation	Apoptosis
P1	I	X	X		X	
P2	I	X	X			
P3	I	X			X	
P4	I	X	X		X	
P5	I	X				
P6	I	X		X		X
P7	I	X				X
P8	I	X	X			X
P9	I	X	X			
P10	II	X	X			
P11	I	X	X			
P12	I	X	X			
P13	I	X	X			
P14	I	X	X			
P15	I	X		X		
P16	I	X		X		
P17	I	X				
P18	I	X				
P19	I	X				
P20	I	X				
P21	I	X				
P22	I	X				
P23	I	X				
P24	I	X				
P25	II	X				
P26	I	X				
P27	II	X				
P28	II	X				

We included 24 WHO grade I and 4 WHO grade II tumor samples and used cultured meningioma cells from these samples for experiments as indicated

### Real-time RT-PCR (qRT-PCR)

RNA was isolated with the TRIZOL reagent (Invitrogen, Carlsbad, CA, USA), digested by DNase, cDNA was synthesized, and quantitative real time RT-PCR (qRT-PCR) was performed [12, 15] using TaqMan primer probes (Applied Biosystems, Foster City, CA, USA): *hGAPDH* (Hs99999905_m1)*, hCXCL16 (Hs00222859_m1), hCXCR6 (Hs00174843_m1).* All 28 different meningioma samples were analyzed by qRT-PCR. The reaction was carried out with the MyiQ™ Single Color Real-time PCR Detection System (BIO-RAD, München, Germany) and fluorescent data were converted into cycle threshold (C_T_) measurements. ∆C_T_ values of each sample were calculated as CT_gene of interest_ – CT _GAPDH._ Relative gene expression was calculated with 2^(normalized CT non-stimulated – normalized CT stimulated)^ = n-fold of control. A ∆C_T_ value of 3.33 corresponds to one magnitude lower gene expression compared to GAPDH (glycerinaldehyde-3-phosphate-dehydrogenase). For each gene, logarithmic linear dependence of C_T_-values from the numbers of copies was verified by using different amounts of cDNA.

### Cell culture

It should be noted that all experiments were performed with cells cultivated from surgical dissected tumors described above. Primary human meningioma cells were cultured in glutamine-supplemented Dulbecco’s modified Eagle’s medium (DMEM) plus 20 % fetal calf serum (FCS) as described previously [15]. Subcultures from 2 to 4 were used. Meningiomas express both mesenchymal and epithelial markers of which EMA (epithelial membrane antigen) and fibronectin can be detected in 90 to 100 % of all investigated solid meningiomas, depending on marker and investigated cohort [16–18]. Thus, identity and purity of meningioma cell cultures were proven routinely by fibronectin (1:100; rabbit polyclonal anti-human fibronectin; Santa Cruz Biotechnology, Santa Cruz, CA), EMA (1:20; mouse monoclonal anti-human EMA; DAKO, Glostrup, Denmark), and glial fibrillary acidic protein (GFAP) (1:200; mouse monoclonal anti-human GFAP, DAKO) immunocytochemistry staining. The primary antibody was omitted for negative controls. All cultured meningiomas showed a positive staining for EMA and fibronectin, but lack GFAP expression.

### Immunocytochemistry (ICC)

For immunocytochemistry examination, cultured primary human meningioma cells were seeded on sterile glass cover slides (50,000 cells/well for routine and CXCL16 staining, 16,000, 64,000 and 160,000/well to investigate the density dependent expression of CXCR6) and grown for two (for density dependent expression analysis) to up to four days in 20 % FCS-supplemented DMEM. In case of stimulations, cells were seeded on glass cover slides (64,000/well), grown for two days and stimulated for 24 h with 10 nM CXCL16 (PeproTech, Hamburg, Germany). Then, cell were washed with phosphate buffered saline (PBS) for three times and fixed with methanol-acetone (1:1; ice-cold) for 10 min and 4 % para-formaldehyde in PBS for 30 min. Non-specific binding was blocked with 0.5 % bovine serum albumin (BSA)/0.5 % glycine in PBS for 60 min. Glass cover slides were incubated with anti-CXCL16 or anti-CXCR6 primary antibody over night at 4 °C (anti-CXCL16, goat polyclonal, 1:100 and anti-CXCR6, mouse monoclonal, 1:100, both R&D Systems, Wiesbaden, Germany). The primary antibody was omitted for negative controls. After washing steps with PBS, glass cover slides were incubated with the Alexa Fluor 555-coupled secondary antibody (red, 1:1,500, donkey anti-goat or anti-mouse IgG, Invitrogen, Life Technologies, Karlsruhe, Germany) for 1 h at 37 °C in darkness. After washing with PBS, nuclei were stained with 4′,6-diamidino-2-phenylindole (DAPI; Molecular Probes/Invitrogen; 1:30,000, 30 min room temperature), washed with PBS (3×) and finally distilled water. After embedding in Immu-Mount (Shandon, Pittsburgh, PA, USA) digital photography was performed using a Zeiss fluorescence microscope and Zeiss camera (Zeiss, Jena, Germany). As a positive control for CXCR6 immunoreactivity, we used LOX melanoma cells transfected with an expression vector for CXCR6 (OriGene, Rockville, MD). Native LOX melanoma cells were a kind gift of Professor Udo Schumacher, University Hospital Hamburg-Eppendorf.

### Binding experiments

Cultured primary human meningioma cells were grown for up to four days on sterile glass cover slides in 20 % FCS-supplemented DMEM, washed with PBS for three times and incubated for 15 min on ice. Meningioma cells were incubated with Cy3-labeled CXCL16 (2 μl diluted in 50 μl PBS) or Cy3-labeled lactalbumin (2 μl diluted in 50 μl PBS) for 60 min on ice*.* For labeling of CXCL16 and lactalbumin, 2 μg protein were incubated with a 4-fold excess of monoreactive Cy3-NHS ester (GE Healthcare, Freiburg, Germany) in 0.2 M NaHCO_3_ buffer, pH 8.4 (total reaction volume 90 μl). After a washing step with PBS, cells were fixed in methanol-acetone (1:1; ice-cold) for 10 min, and washed for three times with PBS. Nuclei were stained with DAPI (see above), and after embedding in Immu-Mount (Shandon) digital photography was performed using a Zeiss fluorescence microscope and Zeiss camera (Zeiss).

### Western blot

Primary human meningioma cells (1.5 × 10^5^) were grown for two days in 20 % FCS-supplemented DMEM, washed in DMEM with 0.5 % FCS for three times (20 min, respectively) and stimulated for 10 min, 20 min and 40 min in the same medium with 10 nM CXCL16 (PeproTech) or for 10 min with epidermal growth factor (EGF; 10 ng/ml; Pepro Tech) or for 20 min with anti-CXCL16 (1 μg/ml; R&D Systems), respectively. In parallel, control cells were kept without stimulation, these were also used to confirm lack of CXCR6 expression on protein level by western blot [6]. Cells were harvested with 1 ml lysis buffer [50 mM TRIS, 100 mM NaCl, 2 mM EDTA, 1 % Triton-X-100, and 1 mM sodium vanadate, 1× Halt™ Phosphatase Inhibitor Cocktail (Thermo Scientific, Bonn, Germany)], 3 μg of protein per sample was loaded on 10 % SDS-polyacrylamide gels for electrophoresis and then transferred to a polyvinylidene difluoride membrane (Hybond™-P PVDF membrane, GE Healthcare, Freiburg, Germany). As a positive control for CXCR6, 50 ng/lane recombinant human CXCR6 (Biozol, Eching, Germany) was applied in a separate lane. To verify CXCL16 expression in membrane isolates, and to exclude that CXCL16 stimulation might induce CXCR6 expression, meningioma cells were grown until confluency, stimulated with 10 nM CXCL16 or not, and cells were harvested and lysed in 5 mH Hepes, pH 7.4. Then, 10 vol.% 200 mM Hepes, 1.4 mM NaCl pH 7.4 was added to each sample, detritus was removed by centrifugation (8 min, 800 xg), and membranes were isolated by centrifugation for 60 min at 14 000 xg. Membrane preparations were solubilized in 20 mM Hepes, 0.14 mM NaCl, pH 7.4, and protein amounts of 5 μg/lane were applied to electrophoresis and blotting as described above. The polyvinylidene difluoride membranes were blocked with 5 % BSA/TBST and incubated with primary antibodies against phospho-p42/44 MAPK (Cell Signaling, Beverly, MA, 1:1,000), phospho-Akt (Cell Signaling, 1:250), CXCR6 (Acris, Hiddenhausen, Germany, 1:250) or CXCL16 (PeproTech, 1:250) at 4 °C overnight. The membranes were incubated with the secondary antibody (1:30,000, donkey anti-rabbit IgG-HRP, Santa Cruz Biotechnology) for 1 h at room temperature, and horseradish peroxidase activity was detected by applying an ECL Advance Western Blotting Detection Kit (GE Healthcare) followed by exposure of the membranes to a sheet of autoradiography film (Hyperfilm™ECL™, GE Healthcare). Equal protein loading was confirmed by either reprobing the membranes with anti-ERK-2 (1:200, Santa Cruz Biotechnology), anti-Akt (1:500, Cell Signaling) or anti-Caveolin-1 (1:200, Santa Cruz Biotechnology) after antibody stripping for 30 min using Reblot Stripping Solution (Millipore, Temecula, CA, USA), or by performing second SDS-polyacrylamide gel with same probes in parallel.

### Proliferation assay

Primary human meningioma cells were plated into 96-well dishes (1,000 cells/ well), grown for two days in 20 % FCS-supplemented DMEM, and stimulated in the same medium with 10 nM CXCL16 (Pepro Tech), 1 μg/ml anti-CXCL16 (R&D Systems) or 10 ng/ml EGF for up to 24 h. In parallel, control wells were kept without stimulation. Proliferation was determined by the measurement of tetrazolium salt WST-1 cleavage (Roche, Mannheim, Germany) and normalized to non-stimulated control (4 individual wells for each stimulus).

### Caspase-3 activity assay

Primary human meningioma cells were plated into 96-well dishes (10,000 cells/ well), grown for two days in 20 % FCS-supplemented DMEM, washed in 37 °C-thermostatted 0.5 % FCS-supplemented DMEM for three times (20 min, respectively), and stimulated in the same medium with 50 μg/ml camptothecin (Sigma Aldrich, Steinheim, Germany), 10 nM CXCL16 (Pepro Tech) or with combination of both for up to 24 h. In parallel, control wells without/with stimulation in DMSO were used. For detection of active caspase-3 amounts, samples were washed in PBS and incubated in 100 μl Homogeneous Caspase 3/7 substrate (Apo-ONE® Homogeneous Caspase-3/7 Assay; Promega, Madison, USA) for 30 min according to the manufacturer’s instruction and as described before [15]. The amounts of active caspase-3 were determined in relation to a caspase 7 standard (Enzo Life Science, Lörrach, Germany), and camptothecin-stimulated wells were set 100 %.

### RNAi silencing

After cultivation of primary human meningioma cells in DMEM plus 20 % FCS in 6-well dishes (150,000 cells/well; for Western Blot experiments) or on sterile glass cover slides (15,000 cells/well; for binding experiments) for 24 h, cells were transfected with siCXCL16 RNA (CXCL16 siRNA ID: s33808; 50 pmol/well; Life Technologies, Darmstadt, Germany) dissolved in a mixture of Opti-MEM Medium and lipofectamine (Life technologies) for 6 h. In parallel a transfection with silencer select negative control siRNA (Life technologies) was performed under same conditions. After transfection cell culture medium was changed and meningioma cells were cultured for another 24 h in DMEM plus 20 % FCS. Then, cells were washed 20 min for three times, respectively, with DMEM plus 0.5 % FCS and afterwards stimulated for 15 min with or without recombinant CXCL16 (10 nM; PeproTech) dissolved in DMEM plus 0.5 % FCS, lysed and applied for Western Blot experiments as described above. For binding experiments primary human meningioma cells were washed with PBS for three times, incubated for 15 min on ice, and stained with Cy3-labeled CXCL16 (2 μl diluted in 50 μl PBS) or Cy3-labeled lactalbumin (2 μl diluted in 50 μl PBS) for 60 min on ice. Fixation of cells and counterstaining of nuclei were performed as described above.

For controlling the knockdown efficiency, RNA of transfected cells were purified in parallel with the PicoPure RNA Isolation Kit (MDS Analytical Technologies, Sunnyvale, CA) according to the manufacturer’s instructions, and qRT-PCR using TaqMan primer probes (Applied Biosystems): *hGAPDH* (Hs99999905_m1) and *hCXCL16 (Hs00222859_m1)* was performed as described above.

## Results

### Cultured human meningioma cells express CXCL16, but not CXCR6

**Fig. 1:**
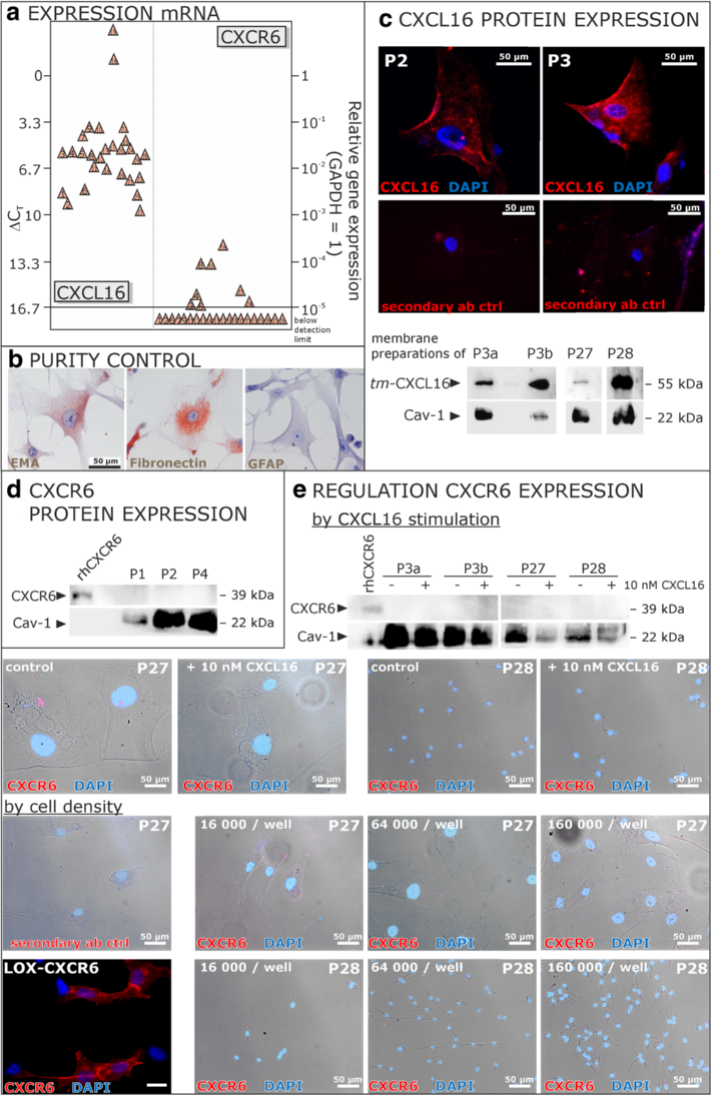
Expression of CXCL16 and its receptor CXCR6 in cells cultivated from human meningiomas. **a** Transcription of CXCL16 and its receptor CXCR6 in human meningioma primary cultures was determined by qRT-PCR (each triangle indicates an individual patient’s sample). Meningioma primary cultures show high mRNA levels of CXCL16, whereas the expression of CXCR6 is hardly detectable or completely lost. **b** The identity and purity of meningioma cultures was assured for every culture by immunocytochemistry on EMA, fibronectin and GFAP, only cultures with positive reactivity for EMA and fibronectin and negative staining for GFAP were used for further experiments (exemplary data). **c** Expression of surface *tm-*CXCL16 on protein level was confirmed by immunocytochemistry using a CXCL16 specific antibody and a red labeled secondary antibody, nuclei were counterstained with DAPI. Furthermore, membrane expression of CXCL16 was proven by western blot using membrane preparations isolated from meningioma cells. Caveolin-1 (Cav-1) served as membrane-specific loading control. **d** Lack of CXCR6 expression was confirmed on protein level by western blot. In parallel to subsequent experiments, cell lysates were analyzed using a CXCR6-specific antibody. Whereas the positive control, human recombinant CXCR6, was sensitively detected (50 ng/lane), cultured meningioma cells did not show any CXCR6 expression. **e** CXCR6 expression was neither induced by CXCL16 stimulation nor by different cell densities. Stimulation with 10 nM recombinant human CXCL16 for 24 h, the maximum stimulation time for subsequent experiments, did not yield an induction of CXCR6 expression as shown by ICC and western blot. Additionally, different cell densities in a range from 16,000 to 160,000 cell/well (representing the cell densities seeded for subsequent experiments) did not influence on the expression of CXCR6 as shown by ICC. Exemplary data of technical (P3 **a** and **b** different cultivation passages) and biological replicates are shown. LOX melanoma cells which were transfected with an expression vector for human CXCR6 served as a positive control to confirm the specificity of the CXCR6 antibody in ICC, recombinant human CXCR6 served as positive control for western blot experiments

We initially measured CXCL16 and CXCR6 mRNA (Fig. 1a) expression in cultured meningioma cells used in our experimental settings. In comparison to CXCL16, which was clearly detectable in all investigated samples, CXCR6 transcription was (nearly) undetectable. To precisely identify the primary cultures as meningioma cells, each culture was routinely immunostained for epithelial membrane antigen (EMA), fibronectin and glial fibrillary acidic protein (GFAP). Examples of this routine staining are shown in Fig. 1b. Only cultures showing positive immunostaining for EMA and fibronectin combined with negative staining for GFAP were used for experiments. On protein level, we could detect CXCL16 expression by immunocytochemistry and western blots of meningioma cell membrane preparations (Fig. 1c) yielding specific bands for the transmembrane (*tm-*)CXCL16. In contrast, CXCR6 could not be detected on protein level by western blot and immunocytochemistry (Fig. 1d and e), and its expression was neither provoked by stimulation with 10 nM CXCL16 nor by cultivation of different cell densities ranging from 16,000 to 160,000 cells per well, which represent the cell densities seeded for subsequent experiments (Fig. 1e). Thus, we concluded that human meningioma cells cultivated from different patients samples substantially express CXCL16 expression while CXCR6 is absent.

### Cultured primary human meningioma cells bind *s-*CXCL16 and show subsequently activated intracellular signaling kinases

**Fig. 2:**
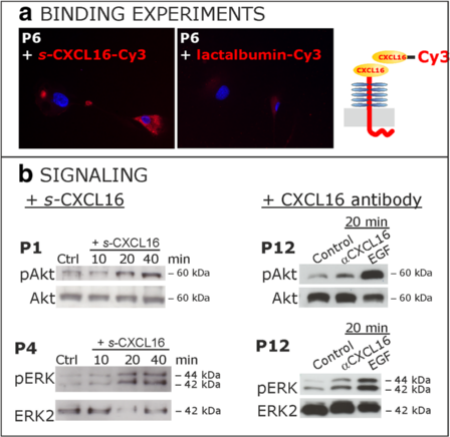
CXCL16 binds to meningioma cells and activates intracellular signaling pathways. **a** Cy3-labeled *s-*CXCL16 binds to primary human meningioma cells, while after incubation with Cy3-labeled lactalbumin binding could not be observed (equal exposure times, representative examples). **b** Stimulation of cultured primary meningioma cells with 10 nM *s-*CXCL16 (left) yields a time-dependent activation of the signal kinases ERK1/2 and Akt as detected by western blot using antibodies for the phosphorylated kinases (pAkt and pERK). Equal protein loading was confirmed by detection of the non-phosphorylated kinases (Akt and ERK2), respectively. This effect was also observed when a CXCL16-targeting antibody (right, ﻿αCXCL16, 1 μg/ml) was used for stimulations. EGF served as positive control, shown are representative examples of at least 3 independent experiments with cultures from different donors

We measured in a next step if cultured CXCR6-negative human primary meningioma cells were able to bind *s-*CXCL16 and transduce signaling effects. In fact, Cy3-labeled *s-*CXCL16 was found to bind to the meningioma cells (Fig. 2a), whereas Cy3-labeled lactalbumin as a negative control did not yield any staining. Next to binding, *s-*CXCL16 was able to induce phosphorylation and thereby activation of both p42/44 extracellular mitogen-activated kinase (ERK1/2) and Akt in a time-dependent manner (Fig. 2b; exemplary results are shown). In all independent experiments, we observed an activation of the respective kinases between 10 and 40 min. However, since primary cultures were obtained from various patients, the time course and intensity of activation differed between cultures. Stimulation with epidermal growth factor (EGF) served as a positive control.

Additionally, in accordance with our recent findings in gliomas [10], application of a specific CXCL16 antibody (αCXCL16) which may also induce the intrinsic activity of *tm-*CXCL16, resulted in phosphorylation of both Akt and ERK1/2 (Fig. 2b, right side) after 20 min.

### Soluble *s-*CXCL16 induces proliferation and rescue from apoptosis

**Fig. 3:**
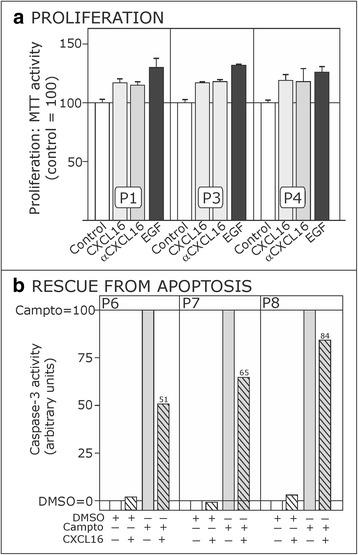
*s-*CXCL16-mediated effects on proliferation and rescue from apoptosis. **a** 10 nM *s-*CXCL16 and 1 μg/ml αCXCL16 promote the proliferation of slowly growing human meningioma cells as measured by MTT activity assay. EGF (10 ng/ml) served as positive control. Control values were set 100 %, and shown are three independent experiments with cultures from different donors, with mean ± SD from 4 technical replicates. **b** When apoptosis was induced in human primary meningioma cells by 50 μg/ml camptothecin (Campto = 100 %), additional incubation with 10 nM CXCL16 could drastically reduce the caspase-3 activity. Shown are three independent experiments with cultures from different donors

To understand which biological consequences were induced after *s-*CXCL16 induced “inverse signaling”, we investigated proliferation effects and whether *s-*CXCL16 could prevent apoptosis in CXCR6-negative but *tm-*CXCL16-positive cultured primary human meningioma cells (Fig. 3).

Indeed, after 24 h stimulation time with *s-*CXCL16, proliferation was induced up to 117 % (P1 and P3; control = 100 %) and 118 % (P4; control = 100 %; Fig. 3a). Comparable results were detectable after application of the specific CXCL16 antibody (αCXCL16). Valuating these results, one should keep in mind that cultured human meningioma cells are large and slow-growing cells. Thus, regarding the fact that the positive control yielded proliferation of meningioma cells within the same range (P1 = 130 %; P3 = 132 %; P4 = 126 %; control = 100 %), CXCL16 stimulation clearly yielded proliferative effects in these cells.

Additionally, CXCL16 was able to reduce caspase-3 activity after camptothecin treatment in CXCR6-negative but *tm-*CXCL16-positive cultured primary human meningioma cells (Fig. 3b). In detail, for sample P6 camptothecin-induced caspase-3 activity (DMSO as control = 0 %; camptothecin dissolved in DMSO = 100 %) was reduced up to 51 %, for P7 up to 65 %, and for P8 up to 84 % after *s-*CXCL16 application.

### CXCL16 knockdown showed specificity of results

**Fig. 4:**
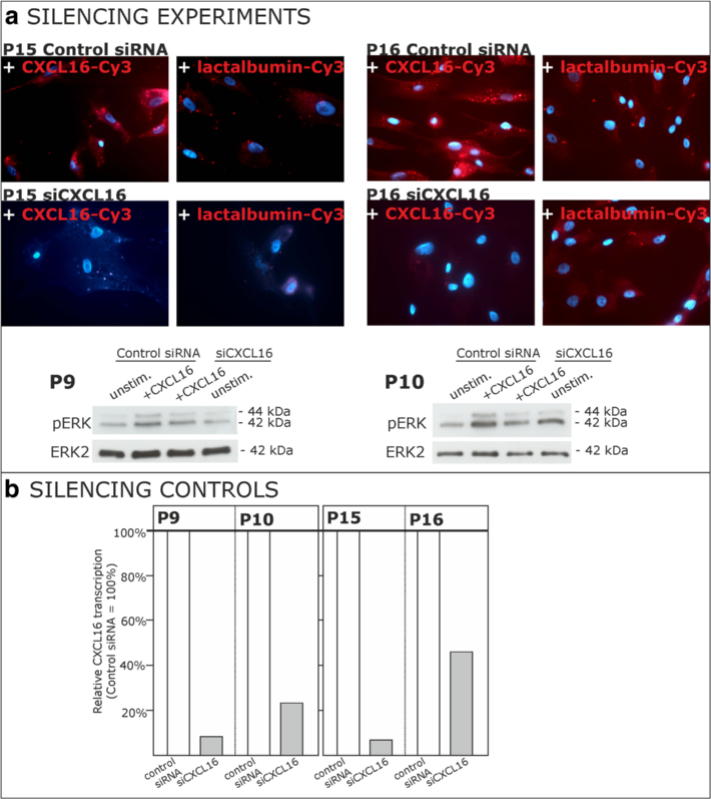
Binding of *s-*CXCL16 and signaling upon stimulation with *s-*CXCL16 depends on the *tm*-CXCL16 level. Expression of the *tm*-CXCL16 was reduced in human meningioma cells by siRNA targeting CXCL16 (for controls, an unspecific control siRNA was used). **a** Knockdown of CXCL16 yielded a drastically reduced binding signal of Cy3-labeled *s-*CXCL16 (upper part). Additionally, we observed reduced activation of the ERK signaling pathway as determined by Western blot on kinase phosphorylation (lower part). **b** The efficient knockdown of CXCL16 was determined by qRT-PCR (bottom right). Shown are 2 independent experiments with meningioma primary cultures from different donors

In a next step, we aimed to show that *tm-*CXCL16 expression is mandatory to the signaling mediated by *s-*CXCL16, to further support the hypothesis of “inverse signaling” in human meningioma cells. In our recent investigations in cultured human glioma cells we proved the specificity of this effect by several different approaches [10]. Due to limited material and characteristics of cultured meningioma cells (e.g. decreased proliferation and increased susceptibility to apoptosis after transfection) we chose binding experiments and kinase activation as model experiments to prove the specificity of the results by CXCL16 knockdown. Results are shown in Fig. 4.

Binding of Cy3-labeled *s-*CXCL16 to *tm-*CXCL16 expressed by cultured human meningioma cells was almost completely abolished after siCXCL16 knockdown in the tumor cells (Fig. 4a, upper part). In contrast, control siRNA transfected meningioma cells were still able to bind *s-*CXCL16. Cy3-labeled lactalbumin served as a negative control for testing unspecific binding background. The expression of CXCL16 as determined by qRT-PCR was reduced to 6.7 % (P15) and to 46 % (P16; Fig. 4b).

Additionally, in relation to meningioma cells transfected with control siRNA, siCXCL16 transfected ones showed reduced phosphorylation and thereby activation of ERK1/2 after *s-*CXCL16 treatment (exemplary results in Fig. 4a, lower part). These results are sustained by the reduction of CXCL16 mRNA expression to 11.5 % (P9) and 28.7 % (P10; Fig. 4b, bottom; control siRNA = 100 %) as determined by qRT-PCR.

Summarized, in cultured primary human meningioma cells the classical CXCL16 receptor CXCR6 is lost, but nevertheless CXCL16 is still able to bind to and transduce signals into the cells resulting in increased proliferation and rescue of apoptosis of the cells. The binding and subsequent cellular activation clearly depends on the expression of *tm-*CXCL16 as shown by siRNA knockdown. Thus, “inverse signaling” of the transmembrane chemokine CXCL16 occurs in cultured primary human meningioma cells, and is involved in progression of human meningiomas.

## Discussion

We recently discovered in highly malignant glioma cells a novel form of para- or autocrine signaling mechanism for transmembrane chemokines which we termed “inverse signaling” [10]. This new signaling concept anticipates that the proteolytically released chemokine domain (*s*-chemokine) specifically binds to its transmembrane counterpart resulting in activation of intracellular kinases and induction of proliferation and anti-apoptosis. Thus, in this novel signaling concept, the transmembrane ligands act as receptors for their soluble counterparts. This signaling concept war sustained by various silencing and transfection experiments. It requires di-/multimerization of the *tm-*chemokine, which can also be induced by di-, but not by monovalent (Fab-fragments) antibodies. We could also show that the intracellular domain (that contains motifs for binding adapter proteins) is essential for signaling. In the present investigation we now describe that cultured primary human meningioma cells exhibited high CXCL16 expression *in vitro*, whereas the CXCL16-specific chemokine receptor CXCR6 was mostly absent. Facing the discrepancy between *tm*-chemokine expression and the lack of the corresponding receptor also in meningioma cells, we hypothesized that the concept of inverse signaling as a broad biological concept may be extended further to more benign tumor entities.

The expression of the chemokine CXCL16 has been reported previously for monocytes/macrophages, B cells, dendritic cells, keratinocytes and endothelial cells [1, 5, 19, 20] while the receptor CXCR6 has been detected quite selectively on activated T cells, NK cells and bone marrow plasma cells [2, 20, 21]. Apart from its physiological expression, CXCL16 is pathologically expressed in cancer cells of different origin including malignant glioma cells and reactive astrocytes *in situ *and in vitro [5–7, 11, 22].

However, although cultured human meningioma cells lack the CXCL16-specific receptor CXCR6, stimulation with *s-*CXCL16 was able to transduce intracellular signaling effects. The recombinant *s-*CXCL16 was able to bind to the cell surface and to induce phosphorylation and thereby activation of the kinases ERK and Akt in a time dependent manner. These results are in a way comparable with previous ones which described CXCL16-mediated ERK activation in schwannoma cells [6, 23], or Akt activation in human aortic smooth muscle cells [24]. However, as the human cultured meningioma cells lack the corresponding receptor CXCR6, a classical receptor mediated signaling could be excluded. To investigate the relevance of *tm*-CXCL16 in the observed signaling process, we performed siCXCL16 knockdown experiments and were able to show that binding to and induction of intracellular signals clearly depends on the expression of *tm-*CXCL16.

In general, the activation of the ERK signal transduction pathway often results in elevated cell growth, and the Akt pathway in anti-apoptotic mechanisms of tumor cells. Therefore, we wanted to know whether stimulation with *s*-CXCL16 could induce these biological responses in cultured primary human meningioma cells. In fact, we could show that both effects – activation of proliferation and rescue of apoptosis – were triggered by *s-*CXCL16 in cultured CXCR6-negative, but *tm-*CXCL16-positive meningioma cells. Additionally, for a more general view on the biological role and regulation of the “inverse signaling” of CXCL16 one should keep in mind that *tm*-chemokines like CXCL16 need to be cleaved by the cell surface proteases ADAM10 and ADAM17 in order to produce their soluble counterparts [5]. Regarding the biological role of CXCL16 in tumors, contrasting its already mentioned effects in glioma and schwannoma cells [5, 6, 21], high CXCL16 expression correlates with good prognosis and increased levels of tumor-infiltrating lymphocytes in renal cancer [25], while in human rectal cancer a downregulation of CXCL16 has been reported [26]. However, pro-tumorigenic effects of CXCL16 have been reported for several other tumors, e.g. breast cancer [9, 27], lung cancer, and is discussed as a promotor of inflammation-associated cancer in general [28], which is underlined by our own findings, that microglia/macrophages isolated from human glioma samples show high expression levels of CXCL16 themselves [29]. Thus, the inverse signaling mechanism of CXCL16 we here describe for meningiomas may occur in a variety of benign and malignant tumors, modulating amongst others the interplay between tumor and immune cells and influencing on tumor progression. Targeting the tumor promoting effects of CXCL16 might be a promising therapy amendment e.g. for atypic meningiomas and other tumors with limited treatment options.

## Conclusion

Summarized, in the present investigations we were able to show that “inverse signaling” takes place in cultured primary human meningioma cells resulting in increased proliferation and rescue of apoptosis of these cells. Thereby, we verified this new mechanism beside its appearance in malignant glioma cells [10] in a different system, and showed that “inverse signaling” seems to be a broad biological process as it is also observable in more benign tumor cells.

## Abbreviations

ADAM: A disintegrin and metalloproteinase
C_T_: Cycle of threshold
DMEM: Dulbecco’s modified Eagle’s medium
EGF: Epidermal growth factor
EMA: Epithelial membrane antigen
FCS: Fetal calf serum
GAPDH: Glycerinaldehyde-3-phosphate-dehydrogenase
GFAP: Glial fibrillary acidic protein
ICC: Immunocytochemistry
qRT-PCR: quantitative reverse-transcription polymerase chain reaction
s: Soluble
tm: transmembrane
WHO: World Health Organization
